# Impact of Hemoglobin Levels and Their Dynamic Changes on the Risk of Atrial Fibrillation: A Nationwide Population-Based Study

**DOI:** 10.1038/s41598-020-63878-9

**Published:** 2020-04-21

**Authors:** Woo-Hyun Lim, Eue-Keun Choi, Kyung-Do Han, So-Ryoung Lee, Myung-Jin Cha, Seil Oh

**Affiliations:** 1 Division of Cardiology, Department of Internal medicine, Seoul National University Boramae Medical Center, Seoul, Republic of Korea; 2 Division of Cardiology, Department of Internal medicine, Seoul National University Hospital, Seoul, Republic of Korea; 3 Department of Biostatistics, College of Medicine, The Catholic University of Korea, Seoul, Republic of Korea

**Keywords:** Atrial fibrillation, Risk factors

## Abstract

Anemia is a risk factor for cardiovascular disease, but its impact on new-onset atrial fibrillation (AF) is unclear. In this study, we investigated the effect of hemoglobin (Hb) levels and their changes on the risk of AF development in the general population of Korea. We retrospectively analyzed a cohort from the Korean National Health Insurance Service database and identified 9,686,314 subjects (49.8% male) without a history of AF, aged ≥40 years, and with Hb levels available for both first (2009–2010) and second (2011–2012) health checkups. These subjects were followed up until 2017 to determine AF development. The presence of anemia (Hb level <13 g/dL in men and <12 g/dL in women) was a significant risk factor for AF development. However, Hb levels showed a U-shaped association with incident AF after adjustment for cardiovascular risk factors. AF incidence was lowest at Hb levels of 14–14.9 g/dL in men and 12–12.9 g/dL in women. Among individuals with Hb levels within normal ranges (13–15.9 g/dL in men and 12–14.9 g/dL in women), both decrease and increase in Hb levels at the second measurement outside the normal ranges showed an elevation of AF risk by 11% and 21% for men and 3% and 36% for women, respectively, compared with those who maintained normal Hb levels. In conclusion, low or high Hb levels are associated with an increased risk of incident AF. This study suggests that maintaining Hb levels within the normal ranges confers a low risk of AF development.

## Introduction

Atrial fibrillation (AF) increased the risk of ischemic stroke and death^1,2^. As the population ages, the prevalence of AF increases, which has a significant impact on public health^3,4^. Therefore, it is important to establish a simple health screening method that can identify people at high risk of AF development.

Anemia is associated with an increased risk of cardiovascular morbidity and mortality in patients with chronic kidney disease, heart failure, or coronary artery disease^5^. In the general population, anemia is also a risk factor for cardiovascular diseases^6^. Several studies suggest that high hematocrit or hemoglobin (Hb) levels are also associated with cardiovascular outcomes^7–9^. However, the impact of anemia or Hb level on AF development in the general population has not been well understood. In this study, we aimed to investigate the effect of Hb levels and their changes on the risk of AF development in the general population of Korea.

## Methods

### Data sources

We used data from the national health claims database provided by the National Health Insurance Service (NHIS). The NHIS is a mandatory health insurance program managed by the Korean government, which covers 97% of the Korean population (approximately 50 million people). The NHIS database includes each subject’s demographic information, diagnoses, their use of inpatient and outpatient services, and prescription claims. Diagnoses are recorded using the International Classification of Disease, 10th Revision, Clinical Modification (ICD-10-CM) codes.

Individuals in the NHIS are recommended to receive standardized health checkups every 2 years. People born in even-numbered years undergo screening every even-numbered years, and those born in odd-numbered years undergo screening in odd-numbered years. Although the resident registration number of each subject in the NHIS is encrypted to protect privacy, it was possible to trace all the claims of the same subject continuously.

This study was exempt from review by the Seoul National University Hospital Institutional Review Board (E-1707-004-864). Informed consent was not obtained because patient records and information were anonymized and de-identified before the analysis.

### Study population and its characteristics

**Figure 1:**
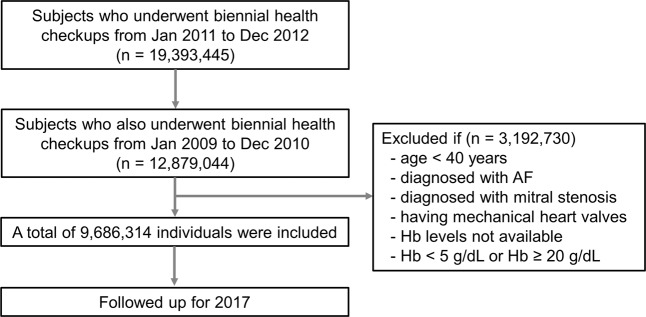
Study population enrollment flow. AF, atrial fibrillation; Hb, hemoglobin.

We identified 12,879,044 Korean residents who had undergone first biennial health checkup provided by the NHIS from January 2009 to December 2010 and second health checkup from January 2011 to December 2012 (index year). Among them, we excluded 3,192,730 subjects who were less than 40 years of age, were diagnosed with AF or mitral stenosis, have mechanical heart valves, and whose Hb levels were unavailable, or <5 or ≥20 g/dL. Finally, 9,686,314 individuals were included and followed up until 2017. Figure 1 and Supplementary Figure 1 show the detailed study flow.

We obtained baseline characteristics of study subjects, including age, sex, body mass index, and comorbidities such as hypertension, diabetes, dyslipidemia, ischemic heart disease, congestive heart failure, stroke, chronic obstructive pulmonary disease, obstructive sleep apnea, and thyroid disease. Definitions of comorbidities are summarized in Supplementary Table 1 and defined as in our previous study^10^. Anemia was defined according to the World Health Organization criteria (Hb level <13.0 g/dL for men and <12.0 g/dL for women)^11^. All subjects underwent two sets of Hb measurements with a 2-year interval.

### Study endpoint

The endpoint of this study was newly diagnosed non-valvular AF (ICD-10-CM codes I480–484, I489) during the follow-up period. Subjects without AF during the follow-up period were censored at the date of their death or at the end of follow-up (December 2017), whichever came first.

### Statistical analysis

Data are presented as mean with standard deviation for normally distributed continuous variables or numbers with percentages for categorical variables. Each male and female population was divided into six groups according to Hb levels at the second health checkup (index year): <12.0, 12.0–12.9, 13.0–13.9, 14.0–14.9, 15.0–15.9, and ≥16.0 g/dL for men and <11.0, 11.0–11.9, 12.0–12.9, 13.0–13.9, 14.0–14.9, and ≥15.0 g/dL for women. AF incidence rates were estimated by dividing the number of AF events by 1000 person-years. Kaplan–Meier curves were plotted for freedom from AF between subjects with anemia and those without anemia and then compared with the log-rank test. Cox proportional hazards models were used to evaluate the risk of AF according to the presence of anemia. Hazard ratios and 95% confidence intervals were calculated. Model 1 was a Cox model without adjustment. Model 2 was a Cox model adjusted for age and sex. Model 3 was model 2 with additional adjustment for body mass index, smoking, alcohol consumption, exercise frequency, and low income. Model 4 was adjusted for comorbidities such as hypertension, diabetes, and dyslipidemia, additionally to model 3. Model 5 was model 4 with additional adjustment for ischemic heart disease, congestive heart failure, stroke, chronic obstructive pulmonary disease, creatinine clearance estimated by Cockcroft-Gault equation, obstructive sleep apnea, and thyroid disease. We also estimated the association of Hb levels with the incidence of AF using Cox proportional hazards models, in which sex was incorporated into Model 2 to 5 as a covariate. A two-sided P-value <0.05 was considered statistically significant. Statistical analyses were performed using SAS version 9.2 (SAS Institute, Cary, NC, USA).

## Results

### Baseline characteristics

A total of 9,686,314 study subjects (4,823,707 men and 4,862,607 women) were analyzed. Table 1 shows the baseline characteristics of the study population by sex. The mean age was 55.6 ± 10.6 years, and 49.8% were male. Current smokers and drinkers were much more frequent in men than women (35.1% vs. 2.2% and 64.1% vs. 18.5%, respectively). Comorbidities were generally similar between both sexes, but anemia was more prevalent in women. The mean Hb levels were 14.8 ± 1.2 g/dL in men and 12.9 ± 1.2 g/dL in women.

**Table 1:** Baseline characteristics of the study population.

Variables*	Male	Female
	(N = 4,823,707)	(N = 4,862,607)
**Demographics**		
Age (years)	54.8 ± 10.7	56.4 ± 10.5
Height (cm)	168.5 ± 6.1	155.2 ± 5.8
Weight (kg)	68.9 ± 9.8	57.2 ± 8.2
Body mass index (kg/m^2^)	24.2 ± 2.9	23.7 ± 3.1
Smoking		
Non-smoker	1,509,123 (31.3)	4,698,860 (96.6)
Ex-smoker	1,620,472 (33.6)	55,928 (1.2)
Current smoker	1,694,112 (35.1)	107,819 (2.2)
**Alcohol drinking**		
Complete or near abstinence	1,729,711 (35.9)	3,964,178 (81.5)
Mild to moderate consumption	2,582,821 (53.5)	875,436 (18.0)
Heavy drinking	511,175 (10.6)	22,993 (0.5)
Regular exercise	2,888,587 (59.9)	2,250,396 (46.3)
Lowest income (quintile)	734,295 (15.2)	1,148,899 (23.6)
**Comorbidities**		
Hypertension	1,763,567 (36.6)	1,586,082 (32.6)
Diabetes	699,665 (14.5)	436,326 (10.3)
Dyslipidemia	1,130,658 (23.4)	1,417,203 (29.1)
Ischemic heart disease	259,908 (5.4)	230,135 (4.7)
Congestive heart failure	47,131 (1.0)	58,550 (1.2)
Stroke	102,503 (2.1)	95,612 (2.0)
Chronic obstructive pulmonary disease	363,256 (7.5)	399,153 (8.2)
Obstructive sleep apnea	7,208 (0.2)	2,405 (0.1)
Thyroid disease	70,158 (1.5)	253,721 (5.2)
Anemia	303,206 (6.3)	825,059 (17.0)
**Measured parameters**		
Hb (g/dL)	14.8 ± 1.2	12.9 ± 1.2
**Hb (g/dL) groups (male/female)**		
<12/<11	89,525 (1.9)	253,027 (5.2)
12–12.9/11–11.9	213,681 (4.4)	572,032 (11.8)
13–13.9/12–12.9	725,279 (15.0)	1,602,146 (33.0)
14–14.9/13–13.9	1,475,190 (30.6)	1,678,000 (34.5)
15–15.9/14–14.9	1,484,460 (30.6)	647,874 (13.3)
≥16/≥15	835,572 (17.3)	109,528 (2.3)
Fasting glucose (mg/dL)	102.5 ± 25.5	97.3 ± 20.6
Systolic blood pressure (mmHg)	125.5 ± 14.2	122.0 ± 15.5
Diastolic blood pressure (mmHg)	78.4 ± 9.7	75.3 ± 9.9
Total cholesterol (mg/dL)	195.2 ± 36.2	200.7 ± 37.1
Creatinine clearance (mL/min)	87.8 ± 37.8	87.7 ± 28.6
**Clinical Outcomes**		
New-onset atrial fibrillation	106,322 (2.2)	80,880 (1.7)
Follow-up duration (years)	5.9 ± 0.9	5.9 ± 0.8

Hb indicates hemoglobin.

*All the P values were <0.001 in the comparison between men and women.

### Risk of AF according to the presence of anemia and baseline Hb levels

**Figure 2:**
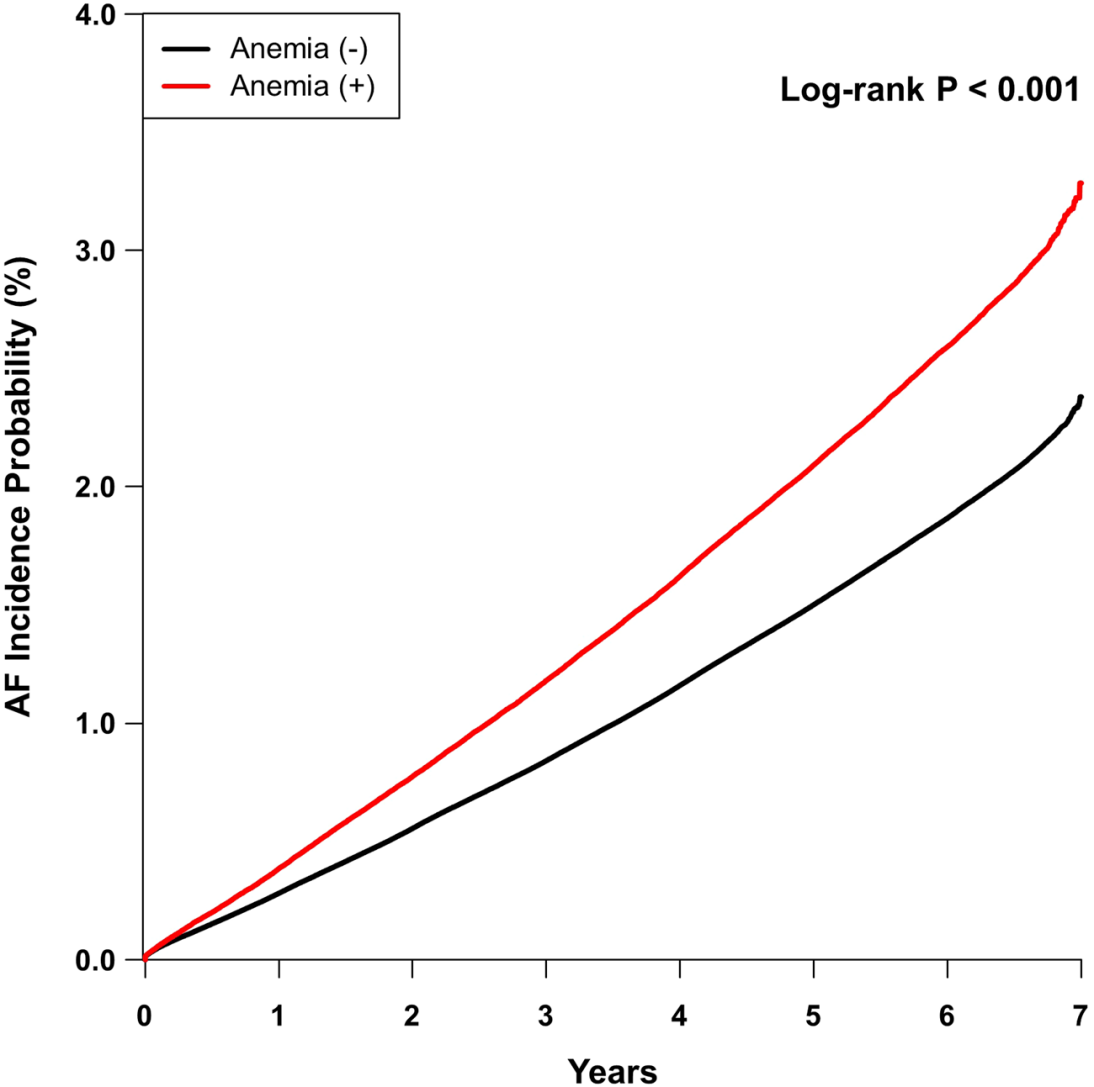
Development of atrial fibrillation (AF) according to the presence of anemia. Kaplan-Meier survival analysis was performed to evaluate the impact of anemia on the incident AF. Subjects with anemia have a significantly increased risk of developing AF compared with those without anemia.

During a mean follow-up period of 5.9 years, 187,202 (1.9%) participants (106,322 men and 80,880 women) developed AF. Figure 2 showed Kaplan-Meier curves for the incidence probability of AF between subjects with anemia and without anemia. Compared with individuals without anemia, the incidence of AF increased significantly in those with anemia (log-rank P < 0.001). After adjustment for age, sex, body mass index, smoking status, alcohol use, exercise, economic status, various cardiovascular comorbidities, and kidney function (Cox proportional hazards model 5), subjects with anemia have a 6% increase in the risk of AF compared with those without anemia (Table 2).

**Table 2:** Incidence and risk of atrial fibrillation according to the presence of anemia.

Anemia	AF cases	Person-years	AF incidence*	Model 1 HR (95% CI)	Model 2 HR (95% CI)	Model 3 HR (95% CI)	Model 4 HR (95% CI)	Model 5 HR (95% CI)
No	158,698	50,285,632	3.156	1.00 (reference)	1.00 (reference)	1.00 (reference)	1.00 (reference)	1.00 (reference)
Yes	28,504	6,499,799	4.39	1.39 (1.38–1.41)^†^	1.08 (1.06–1.09)^†^	1.13 (1.11–1.14)^†^	1.11(1.10–1.12)^†^	1.06 (1.05–1.08)^†^

AF indicates atrial fibrillation; CI, confidence interval; HR, hazard ratio.

*Incidence rates were calculated per 1,000 person-years. ^†^P < 0.001

Model 1: Cox proportional hazards model without adjustment. Model 2: Model 1 with adjustment for age and sex. Model 3: Model 2 with additional adjustment for body mass index, smoking, alcohol consumption, exercise frequency, and low income. Model 4: Model 3 with additional adjustment for hypertension, diabetes, and dyslipidemia. Model 5: Model 4 with additional adjustment for ischemic heart disease, congestive heart failure, stroke, chronic obstructive pulmonary disease, estimated creatinine clearance, obstructive sleep apnea, and thyroid disease.

**Figure 3:**
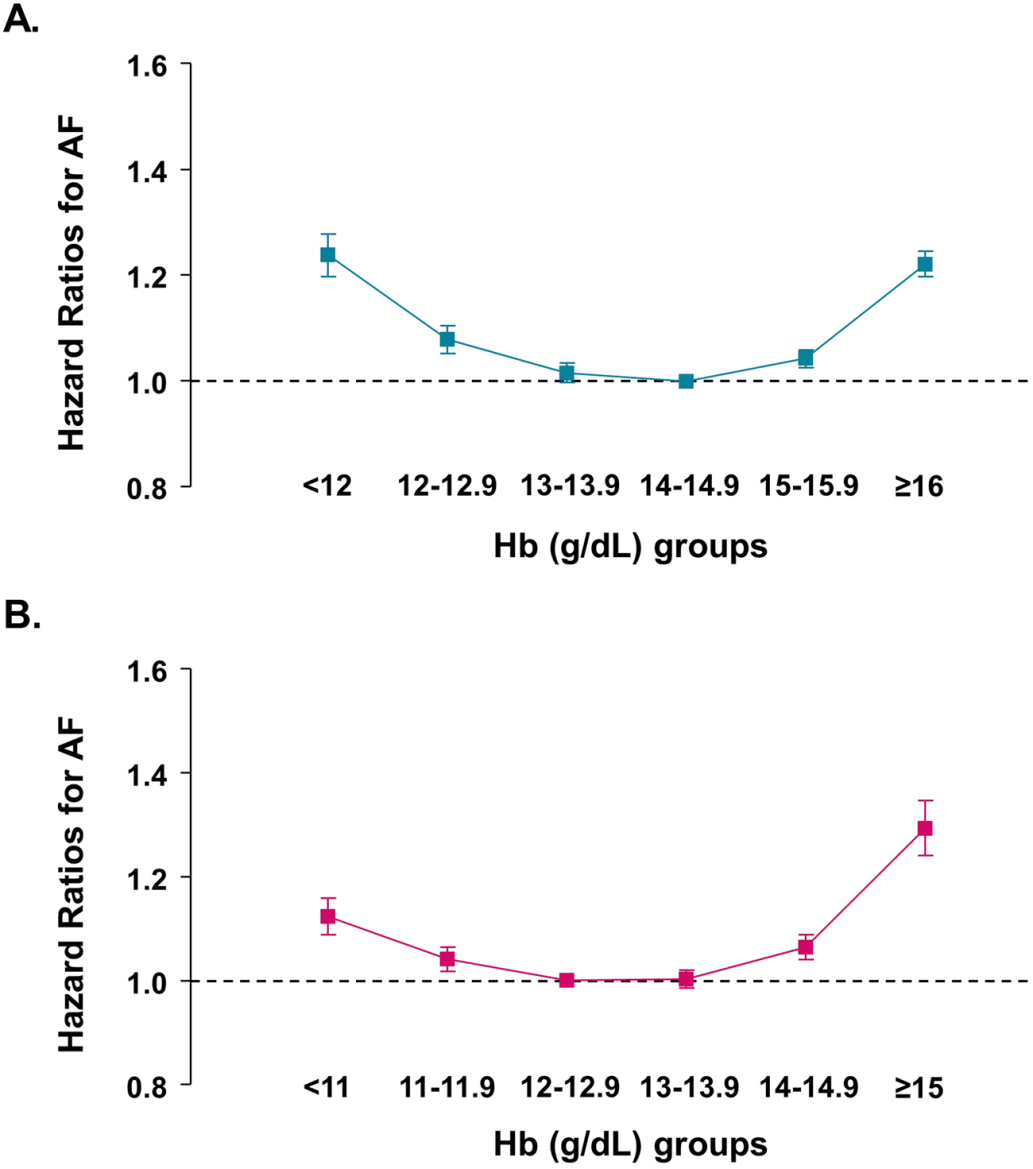
Association between hemoglobin level and risk of atrial fibrillation by sex. (**A**) Men; (**B**) women. The X-axis represents hemoglobin (Hb) groups, while the Y-axis the hazard ratios with reference to Hb levels of 14–14.9 g/dL in men and 12–12.9 g/dL in women. Squares represent hazard ratios, and vertical lines 95% confidence intervals. Hazard ratios were calculated using a Cox proportional hazards model adjusted for age, body mass index, smoking, alcohol consumption, exercise frequency, low income, hypertension, diabetes, dyslipidemia, ischemic heart disease, congestive heart failure, stroke, chronic obstructive pulmonary disease, estimated creatinine clearance, obstructive sleep apnea, and thyroid disease (Model 5 in Table 3).

Table 3 presents the incidence and risk of AF based on Hb levels. The crude AF incidence in men was highest in the group with the lowest Hb level (<12 g/dL) and gradually decreased as Hb level increased up to 16 g/dL. In women, the crude AF incidence was high in both groups with the lowest and highest Hb levels (<11 and ≥15 g/dL, respectively). After Cox proportional hazards analysis (model 5), both low and high Hb levels were associated with an increased risk of AF in both sexes. AF risk was lowest in Hb levels of 14–14.9 g/dL in men and 12–12.9 g/dL in women (reference groups). In other words, Hb levels showed a U-shaped relationship with the risk of AF development, and individuals with higher Hb levels have a greater risk than those with lower Hb levels (Figure 3). In men with Hb level ≥16 g/dL, the risk of AF increased by 22% compared with the reference group, and in women with Hb ≥15 g/dL, the risk increased by 29%.

**Table 3:** Incidence and risk of atrial fibrillation according to the hemoglobin concentrations in each sex.

Hb Groups	AF cases	Person-years	AF incidence*	Model 1 HR (95% CI)	Model 2 HR (95% CI)	Model 3 HR (95% CI)	Model 4 HR (95% CI)	Model 5 HR (95% CI)
**Male**								
<12	4,558	473,386	9.63	2.75 (2.67–2.84)^†^	1.31 (1.27–1.35)^†^	1.39 (1.34–1.43)^†^	1.34 (1.30–1.38)^†^	1.24 (1.20–1.28)^†^
12–12.9	8,235	1,208,864	6.81	1.94 (1.89–1.98)^†^	1.10 (1.07–1.13)^†^	1.14 (1.11–1.17)^†^	1.12 (1.09–1.14)^†^	1.08 (1.05–1.11)^†^
13–13.9	19,853	4,224,888	4.70	1.33 (1.31–1.36)^†^	1.02 (1.00–1.04)^‡^	1.04 (1.02–1.06)^†^	1.03 (1.01–1.05)^†^	1.02 (1.00–1.04)^§^
14–14.9	30,593	8,678,822	3.53	1.00 (reference)	1.00 (reference)	1.00 (reference)	1.00 (reference)	1.00 (reference)
15–15.9	26,872	8,740,244	3.07	0.87 (0.86–0.89)^†^	1.05 (1.04–1.07)^†^	1.03 (1.02–1.05)^†^	1.04 (1.02–1.05)^†^	1.04 (1.03–1.06)^†^
≥16	16,211	4,892,803	3.31	0.94 (0.92–0.96)^†^	1.27 (1.24–1.29)^†^	1.21 (1.19–1.24)^†^	1.21 (1.19–1.24)^†^	1.22 (1.20–1.25)^†^
**Female**								
<11	4,654	1,464,439	3.18	1.15 (1.11–1.19)^†^	1.18 (1.14–1.21)^†^	1.22 (1.18–1.26)^†^	1.19 (1.16–1.23)^†^	1.12 (1.09–1.16)^†^
11–11.9	11,057	3,353,110	3.30	1.19 (1.16–1.22)^†^	1.06 (1.03–1.08)^†^	1.08 (1.05–1.10)^†^	1.06 (1.04–1.09)^†^	1.04 (1.02–1.07)^†^
12–12.9	26,177	9,442,045	2.77	1.00 (reference)	1.00 (reference)	1.00 (reference)	1.00 (reference)	1.00 (reference)
13–13.9	25,652	9,867,829	2.60	0.94 (0.92–0.96)^†^	1.01 (0.99–1.03)^§^	0.99 (0.97–1.01)^§^	0.99 (0.97–1.01)^§^	1.00 (0.99–1.02)^§^
14–14.9	10,842	3,799,451	2.85	1.03 (1.01–1.05)^†^	1.10 (1.08–1.13)^†^	1.06 (1.03–1.08)^†^	1.05 (1.03–1.07)^†^	1.06 (1.04–1.09)^†^
≥15	2,498	639,550	3.91	1.41 (1.36–1.47)^†^	1.41 (1.35–1.46)^†^	1.31 (1.26–1.37)^†^	1.28 (1.23–1.34)^†^	1.29 (1.24–1.35)^†^

AF indicates atrial fibrillation; CI, confidence interval; Hb, hemoglobin; HR, hazard ratio.

*Incidence rates were calculated per 1,000 person-years. ^†^P < 0.001, ^‡^P < 0.05, ^§^P ≥ 0.05

Model 1: Cox proportional hazards model without adjustment. Model 2: Model 1 with adjustment for age. Model 3: Model 2 with additional adjustment for body mass index, smoking, alcohol consumption, exercise frequency, and low income. Model 4: Model 3 with additional adjustment for hypertension, diabetes, and dyslipidemia. Model 5: Model 4 with additional adjustment for ischemic heart disease, congestive heart failure, stroke, chronic obstructive pulmonary disease, estimated creatinine clearance, obstructive sleep apnea, and thyroid disease.

### Risk of AF according to the dynamic change in Hb levels

Table 4 demonstrates the association between dynamic change in Hb levels over a 2-year interval and risk of AF development. Among subjects with normal Hb levels (13–15.9 g/dL in men and 12–14.9 g/dL in women) at the first health checkup, both decrease and increase in Hb levels at the second measurement outside the normal ranges elevated AF risk by 11% and 21% for men and 3% and 27% for women, respectively, compared with those who maintained normal Hb levels (reference groups). Among those with anemia, increase in Hb levels above normal ranges (≥16 g/dL in men and ≥15 g/dL in women) at the second evaluation significantly increased AF risk by 37% for men and 9% for women, compared with the reference groups. Individuals maintaining high Hb levels above normal ranges in 2 years had a 21% higher risk of AF in men and 36% in women. Taken together, not only subjects with low or high Hb levels but also those with dynamic change in Hb levels outside the normal range showed a higher risk of AF compared with subjects maintaining normal Hb levels in 2 years.

**Table 4:** Risk of atrial fibrillation according to the dynamic change in hemoglobin levels.

1^st^ Hb	2^nd^ Hb	AF cases	Person-years	AF incidence*	HR** (95% CI)
**Male**					
<13	<13	5,491	629,066	8.73	1.14 (1.10–1.17)^†^
	13–16	5,267	900,938	5.85	1.09 (1.06–1.13)^†^
	≥16	170	29,794	5.71	1.37 (1.18–1.60)^‡^
13–15.9	<13	7,073	1,022,907	6.91	1.11 (1.08–1.14)^†^
	13–16	64,562	18,498,391	3.49	1.00 (reference)
	≥16	8,476	2,476,163	3.42	1.21 (1.18–1.24)^†^
≥16	<13	229	30,277	7.56	1.45 (1.27–1.65)^‡^
	13–16	7,489	2,244,626	3.34	1.08 (1.05–1.10)^†^
	≥16	7,565	2,386,845	3.17	1.21 (1.18–1.24)^†^
**Female**					
<12	<12	7,841	2,461,947	3.18	1.09 (1.06–1.11)^†^
	12–15	7,451	2,705,094	2.75	1.04 (1.01–1.06)^†^
	≥15	67	24,670	2.72	1.09 (0.85–1.38)^§^
12–14.9	<12	7,812	2,341,077	3.34	1.03 (1.01–1.06)^‡^
	12–15	53,712	19,973,006	2.69	1.00 (reference)
	≥15	1,778	468,250	3.80	1.27 (1.21–1.33)^†^
≥15	<12	58	14,525	3.99	1.03 (0.79–1.33)^§^
	12–15	1,508	431,225	3.50	1.10 (1.04–1.16)^‡^
	≥15	653	146,630	4.45	1.36 (1.26–1.47)^‡^

AF indicates atrial fibrillation; CI, confidence interval; Hb, hemoglobin; HR, hazard ratio.

*Incidence rates were calculated per 1,000 person-years.

**HR was calculated by Cox proportional hazards model 5 as in Table 3. ^†^P < 0.001, ^‡^P < 0.05, ^§^P ≥ 0.05.

## Discussion

To the best of our knowledge, this is the largest population-based study to evaluate the impact of Hb levels on the risk of AF development. Our study showed that (1) anemia was a risk factor for AF development, (2) Hb levels showed a U-shaped relationship with the risk of incident AF after adjusting for various demographic and cardiovascular risk factors, and (3) maintaining Hb levels within normal range conferred the lowest risk of AF, while maintaining high Hb levels or increase to high Hb levels posed the highest risk of AF.

There are only a few previous studies on anemia and incident AF. In a retrospective cohort study of 2,873 elderly patients, anemia was not associated with new-onset AF during the 2-year follow-up^12^. However, the size of the cohort was small, and the follow-up duration was not long enough to determine the difference in incident AF. In a Japanese population-based cohort study of 132,250 subjects with a mean follow-up period of 13.8 years, lower Hb levels and chronic kidney disease were independently associated with an increased risk of incident AF^13^. The authors divided the total population into three groups according to Hb levels (<13, 13–14.9, 15–17.9 g/dL for men, <11, 11–12.9, 13–15.9 g/dL for women), and the third group was used as a reference. Consequently, the third reference group included people with both normal and high Hb levels. In contrast, we divided the total population into six Hb groups and found that both low and high Hb levels are independent risk factors for incident AF and high Hb levels increase AF risk more strongly. There are another two studies with a rather specific study population. One is a retrospective, nested case-control study that evaluated whether parameters of the blood count are associated with the development of new-onset AF in 198 patients with acute myocardial infarction^14^. Among subjects with acute myocardial infarction, those who developed AF showed a significantly higher hemoglobin level compared with those without AF (14.2 g/dl, IQR 12.4–15 vs. 12.9 g/dl, IQR 11.7–13.8; P < 0.001). The other is a nationwide cohort study of aplastic anemia in Taiwan^15^. Patients with aplastic anemia had a higher risk of incident AF compared with the general population after controlling for the competing risk of death (HR 1.21, 95% CI 0.97–1.50). However, the previous two studies do not represent the general population.

Since Hb values are variable with time, we analyzed the risk of AF by dynamic changes in Hb levels. Our study showed that subjects maintaining Hb levels within normal ranges had the lowest risk of new-onset AF. However, those who had maintained high Hb levels or whose Hb levels had increased to high levels in 2 years had the highest AF risk. Among those with high Hb levels, change in Hb levels to normal ranges resulted in a reduction in AF risk. These results suggest that AF risk may be lowered by controlling the conditions that can increase Hb levels, such as cigarette smoking or chronic obstructive pulmonary disease as well as by avoiding anemia.

Chronic anemia can cause many physiologic changes to the circulatory system. Low blood viscosity and hypoxic vasodilation contribute to low peripheral resistance^16^. Along with increased sympathetic activity, chronic anemia leads to an increment in cardiac output, leading to left ventricular (LV) remodeling^17^. On the contrary, an increased number of red blood cells is related to high blood viscosity and increased peripheral resistance, leading to elevated afterload^18^. Therefore, LV remodeling may also develop in patients with high hematocrit levels resulting from chronic hypoxic conditions such as chronic obstructive pulmonary disease or heavy smoking^19^. This relationship between hematocrit levels and LV remodeling is well demonstrated in the MONICA Augsburg substudy^20^. In this study, hematocrit levels showed a U-shape relationship with LV mass and wall thickness in the general population. Accordingly, LV remodeling is more likely to develop in subjects with low or high Hb levels. This might be the reason why low and high Hb levels increase the risk of AF, as LV hypertrophy itself is an independent risk factor for incident AF^21^.

Structural and functional left atrial (LA) remodeling is usually a consequence of impaired LV function and elevated LV filling pressure. However, LA remodeling seems to begin at an early stage of LV remodeling or LV diastolic dysfunction^22,23^. Katayama *et al*. showed that increased LV mass index and low Hb concentration was independently associated with LA enlargement in patients with normal LV systolic function^24^. This result suggests that hemodynamic change by low or high Hb levels can affect LA remodeling and the development of AF before noticeable change such as LV hypertrophy or systolic dysfunction occurs.

In our study, the risk of AF increased in subjects with anemia and low-normal Hb levels (Hb level <13 and 13–13.9 g/dL in men, and <12 and 12–12.9 g/dL in women) compared with the reference groups. However, the absolute risk was higher in men than women (24% and 8% in men, and 12% and 4% in women, respectively). This difference may additionally explain the sex-difference in AF incidence. In Korea, the estimated incidence of AF is 1.17 times higher in men than in women^4^. The difference in AF risk between sex could be explained by differences in risk factors, genetics, cardiac structure, and hormones^25^.

### Study limitations

This study has several limitations. First, selection bias could exist, although the number of our study subjects was more than 9 million. The NHIS provides health checkups every 2 years to all health insurers, but only half of the subjects received this health checkup. Therefore, the study population is likely to include those who maintain a healthy lifestyle or are more concerned about their health. Second, the study population was not regularly followed during the study period, so there is a possibility of bias of lost to follow-up. Considering all the Korean population were covered by mandatory national health insurance, most of the subjects could be tracked in the Korean National Health Insurance Service database except for immigration. Third, the definitions of diseases including AF were manipulated using claims data from the NHIS database. The misclassification of diagnostic codes could be the cause of bias, resulting in an underestimation or overestimation. Nevertheless, the diseases were well defined and repeatedly validated in our previous studies^10,26,27^. Lastly, we were unable to identify the cause of anemia in subjects with anemia and the reasons for high Hb levels in some individuals. However, we adjusted for various factors that may cause low or high Hb levels, such as age, smoking status, ischemic heart disease, congestive heart failure, chronic obstructive pulmonary disease, and kidney function estimated by creatinine clearance.

## Conclusions

Anemia is a risk factor for incident AF. Both low and high Hb levels are associated with an increased AF risk. Our study suggests that maintaining Hb levels within normal ranges would lower the risk of AF development.

## Supplementary information

Supplementary Information.
