# Supplementary material for: Impact of Hemoglobin Levels and Their Dynamic Changes on the Risk of Atrial Fibrillation: A Nationwide Population-Based Study
Source: Sci Rep. 2020 Apr 21;10:6762. doi: 10.1038/s41598-020-63878-9 (PMC7174343; doi:10.1038/s41598-020-63878-9)
Supplement: Supplementary file 1 — Supplementary Information. [file 41598_2020_63878_MOESM1_ESM.pdf]

Impact of Hemoglobin Levels and Their Dynamic Changes on the Risk of Atrial Fibrillation: A Nationwide Population-Based Study

Woo-Hyun Lim, MD[1], Eue-Keun Choi, MD, PhD[2], Kyung-Do Han, MS[3], So-Ryoung Lee, MD[2], Myung-Jin Cha, MD[2], Seil Oh, MD, Ph.D., FHRS[2]

[1]Division of Cardiology, Department of Internal medicine, Seoul National University Boramae Medical Center, Seoul, Republic of Korea; [2]Division of Cardiology, Department of Internal medicine, Seoul National University Hospital, Seoul, Republic of Korea; [3]Department of Biostatistics, College of Medicine, The Catholic University of Korea, Seoul, Republic of Korea;

Running Title: hemoglobin level and atrial fibrillation

Corresponding Author:

Eue-Keun Choi, MD, PhD

Department of Internal Medicine, Seoul National University Hospital

101 Daehak-ro, Jongno-gu, Seoul, 03080, Republic of Korea

Phone +82-2-2072-0688/Fax +82-2-762-9662

E-mail: choiek17@snu.ac.kr

Supplementary Information

I. Supplementary Tables

Supplementary Table 1. Definition of comorbidities and clinical outcomes

Diagnosis	ICD-10-CM code	Diagnostic definition
**Atrial fibrillation**	I480-484, I489	Admission or outpatient department≥1
**Valvular atrial fibrillation**	I050, I052, I059, Z952-Z954	Admission or outpatient department≥1
**Hypertension**	I10-I13, I15	Admission≥1 or outpatient department≥2<br>Minimum 1 prescription of anti-hypertensive medications (thiazide, loop diuretics, aldosterone antagonist, alpha-/beta-blocker, calcium-channel blocker, angiotensin-converting enzyme inhibitor, and angiotensin II receptor blocker).
**Diabetes mellitus**	E11-E14	Admission≥1 or outpatient department≥2<br>Minimum 1 prescription of anti-diabetic medications (sulfonylureas, metformin, meglitinides, thiazolidinediones, dipeptidyl peptidase-4 inhibitors, α-glucosidase inhibitors and insulin).
**Dyslipidemia**	E78	Admission or outpatient department≥1
**Ischemic heart disease**	I20-25	Admission or outpatient department≥1
**Congestive heart failure**	I50	Admission or outpatient department≥1
**Stroke**	I63, I64	Admission≥1 and brain imaging (CT or MRI) ≥1
**Chronic obstructive pulmonary disease**	J41-44	Admission or outpatient department≥1

**Obstructive sleep apnea**	G473	Admission or outpatient department≥1
**Thyroid disease**		
Hypothyroidism	E02-E03	Admission or outpatient department≥1<br>Minimum 1 prescription of thyroid hormones
Hyperthyroidism	E05	Admission or outpatient department≥1<br>Minimum 1 prescription of antithyroid medications (propylthiouracil and methimazole)

Abbreviation: ICD-10-CM, International Classification of Diseases, Tenth Revision, Clinical Modification.

II. Supplementary Figures

Supplementary Figure 1. Timeline of the study

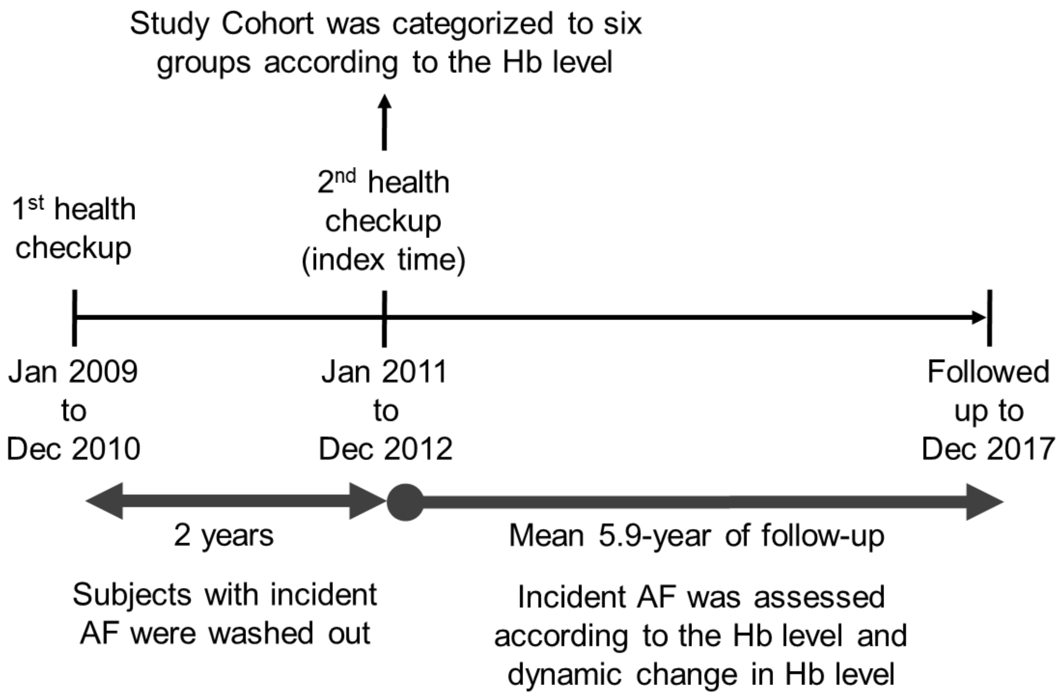

Dynamic change of Hb levels between 2 years

1st Hb level		2nd Hb level
Anemic	→	Anemic, normal, upper normal
Normal	→	Anemic, normal, upper normal
Upper normal	→	Anemic, normal, upper normal

Abbreviations: AF, atrial fibrillation; Hb, hemoglobin
